# Network pharmacology analysis and experimental validation elucidate the protective mechanisms of BYHWD against hypoxia/reoxygenation-induced endothelial cell injury

**DOI:** 10.3389/fmed.2025.1704873

**Published:** 2025-12-19

**Authors:** Yan Shi, Qingnan Zhu, Yue Zhou, Qing Guan, Qingsi Wen, Yu Sun, Zewen Yan, Yuye Li, Yangjianing Zhao, Lu Liu, Hongli Lin, Dapeng Wang

**Affiliations:** 1 Department of Nephrology, The First affiliated Hospital, Dalian Medical University, Dalian, China; 2 Institute of Integrative Medicine, Dalian Medical University, Dalian, China

**Keywords:** BYHWD, hypoxia/reoxygenation, endothelial cells, IRI, acute kidney injury, network pharmacology

## Abstract

**Objective:**

Acute kidney injury (AKI) is a critical clinical condition with high mortality, and specific therapeutic drugs are currently lacking. Although Buyang Huanwu Decoction (BYHWD) has shown clinical efficacy against AKI, its underlying mechanisms remain unclear. This study integrated network pharmacology, *in vitro* experiments, and animal models to systematically elucidate the potential targets and signaling pathways of BYHWD in treating AKI, and to validate its protective effects on hypoxia/reoxygenation (H/R)-induced endothelial cell injury and renal ischemia-reperfusion injury (IRI) *in vivo*.

**Methods:**

Active components and putative targets of BYHWD were screened using network pharmacology, and their intersections with AKI-related disease targets were identified. Protein-protein interaction (PPI) analysis, Gene Ontology and Kyoto Encyclopedia of Genes and Genomes (KEGG) enrichment analyses, and molecular docking were performed. An *in vitro* H/R injury model was established using human umbilical vein endothelial cells (HUVECs). Cell counting kit-8 (CCK-8), trypan blue staining, flow cytometry, and Western blot were applied to assess the effects of BYHWD-containing serum on cell proliferation, apoptosis, oxidative stress, and the expression of proteins related to the VEGFRII/PI3K/AKT/FOXO1 pathway. For *in vivo* validation, a rat model of AKI was established via renal IRI. Rats were randomly divided into sham, IRI model, and BYHWD treatment groups. Renal function was assessed by measuring serum creatinine (SCr) and blood urea nitrogen levels. Renal histopathological changes were evaluated by hematoxylin and eosin (H&E) and Periodic Acid-Schiff staining.

**Results:**

Network pharmacology identified 133 active components in BYHWD and 210 overlapping drug-disease targets. PPI analysis revealed hub genes including VEGFA, AKT1, IL6, and TP53. KEGG enrichment analysis highlighted the PI3K-AKT signaling pathway as a central pathway. Molecular docking demonstrated stable binding of luteolin and quercetin to VEGFA. *In vitro* experiments confirmed that BYHWD-containing serum increased HUVECs viability, inhibited apoptosis, reduced ROS levels, and modulated the protein expression of Bax/Bcl-2, MCP-1, α-SMA, and CD31. Furthermore, BYHWD activated VEGFRII and the downstream PI3K/AKT/FOXO1 pathway. In animal experiments, BYHWD treatment significantly ameliorated renal dysfunction in IRI-induced AKI rats, as evidenced by decreased SCr and BUN levels. Histopathological examination showed that BYHWD attenuated tubular injury, necrosis, and cast formation.

**Conclusion:**

BYHWD may alleviate H/R-induced endothelial cell injury by suppressing oxidative stress and apoptosis through active components such as luteolin and quercetin, which target key genes including VEGFA and AKT1, thereby activating the PI3K/AKT/FOXO1 signaling pathway. This study provides integrated experimental evidence from network pharmacology, *in vitro*, and *in vivo* studies, supporting the use of BYHWD in AKI treatment.

## 1 Introduction

AKI, characterized by rapid decline in renal function, represents a major public health challenge with high morbidity and mortality. A multinational prospective study by the International Society of Nephrology involving 289 centers across 322 countries revealed AKI incidence rates of 39 and 53% in adults and children, respectively (1), contributing to approximately 1.7 million annual deaths worldwide (2). Recent epidemiological studies and meta-analyses demonstrate that 20–50% of AKI survivors develop chronic kidney disease (CKD), with clinical evidence suggesting even mild AKI with apparent recovery may predispose to CKD progression (3, 4). Notably, AKI-CKD transition constitutes a critical pathway to end-stage renal disease (ESRD) (5, 6).

Despite decades of research, AKI pathogenesis remains incompletely understood, involving intricate interactions among tubular, microvascular, and inflammatory components. Acute insults typically induce tubular epithelial cell death, endothelial dysfunction, and leukocyte infiltration (3, 7), while sustained injury leads to tubular degeneration, inflammation, and renal fibrosis (8). Proximal tubular cell (PTC) loss, a hallmark of AKI-CKD transition, is both a consequence and driver of fibrogenesis (9). PTCs (Proximal Tubular Cells), composed of capillary endothelial cells and pericytes, rely on pericyte-endothelial interactions to maintain microvascular integrity. During AKI, endothelial apoptosis and pericyte detachment/dedifferentiation disrupt this architecture, culminating in capillary rarefaction and CKD progression (10, 11). These findings underscore PTC preservation as a pivotal strategy to mitigate AKI-CKD transition.

Vascular endothelial growth factors (VEGFs), through binding to VEGFR-2, orchestrate endothelial survival, proliferation, and angiogenesis via ERK (Extracellular Signal-Regulated Kinase) and PI3K/AKT signaling pathways. PI3K/AKT activation enhances vascular permeability and sustains epithelial viability (12), while stimulating renal microvascular endothelial cell (RMVEC) proliferation to promote PTC regeneration (13). FOXO1 (Forkhead Box Protein O1) transcription factor emerges as a key regulator in AKI pathogenesis. Reduced Klotho exacerbates tacrolimus-induced nephropathy through PI3K/AKT/FOXO1 signaling (14), where AKT-mediated FOXO1 phosphorylation modulates apoptosis via downstream effectors (e.g., Bcl-2/Bax). Experimental evidence confirms the PI3K/AKT/FOXO1 axis as a master regulator of microvascular homeostasis, with pathway activation promoting angiogenesis through FOXO1 phosphorylation (15).

The unique role of traditional Chinese medicine (TCM) in preventing and treating AKI has been increasingly recognized by modern medical science. According to TCM theory, the pathogenesis of AKI centers on “deficiency of qi and blood stasis, with turbid toxins causing obstruction,” and the corresponding therapeutic approach emphasizes “tonifying qi and activating blood circulation, as well as removing toxins and unblocking the meridians” (16). This theoretical framework, particularly the principle of addressing “blood stasis” and unblocking collaterals, provides a strong rationale for investigating classical formulas designed for these purposes in the context of AKI, which is characterized by microvascular dysfunction, coagulation abnormalities, and tissue ischemia.

BYHWD, a classical formula devised by the Qing Dynasty physician Wang Qingren in his work Corrections on Errors in Medical Classics, consists of seven medicinal herbs: Astragalus membranaceus (Huangqi), Angelica sinensis (Danggui), Paeonia lactiflora (Chishao), Carthamus tinctorius (Honghua), Lumbricus (Dilong), Prunus persica (Taoren), and Carthamus tinctorius (Honghua). This formula is known for its effects of reinforcing qi and activating blood circulation, resolving stasis, and dredging the meridians. It is widely used in clinical practice for the prevention and treatment of cardiovascular and cerebrovascular diseases (17).

The formula design follows the principle that “sufficient qi promotes smooth blood flow,” with Astragalus membranaceus serving as the sovereign herb at a dose of 50–100 g. Angelica sinensis acts as the minister herb to promote blood circulation, supported by four blood-activating and stasis-resolving herbs—Paeonia lactiflora, Carthamus tinctorius, Prunus persica, and Carthamus tinctorius—as adjuvant herbs. Lumbricus serves as the guide herb to unblock the meridians. Together, these components form a formula characterized by “prioritizing qi supplementation, complemented by stasis resolution” (18). It is widely used in clinical practice for the prevention and treatment of cardiovascular and cerebrovascular diseases, conditions often sharing microcirculatory and ischemic pathomechanisms with AKI (18).

The therapeutic promise of BYHWD is believed to lie in the synergistic interaction of its multiple constituents, aligning with the polypharmacological nature of TCM formulas. While the multi-target regulatory mechanisms of BYHWD in AKI remain incompletely characterized, foundational investigations suggest its potential. For instance, Chen and Luo et al. demonstrated that BYHWD could protect against cerebral ischemia-reperfusion injury via the PI3K/Akt pathway, a key pathway also critically involved in AKI (19–22). Furthermore, studies on its isolated active constituents provide supporting insights: Astragaloside IV confers protection against sepsis-induced renal tubular injury by activating the PI3K/AKT pathway (23, 24); Ligustrazine ameliorates microcirculatory dysfunction and suppresses oxidative stress responses (25). Paeoniflorin attenuates H/R-induced AKI by reducing oxidative damage via the Nrf2/HO-1 pathway and inhibiting apoptotic processes (26, 27); Angelica sinensis extracts decelerate renal fibrotic progression and preserve the structural integrity of renal tubular epithelial cells (28). Hydroxysafflor yellow A, by activating the AKT/GSK-3β/Fyn-Nrf2 axis, emerges as a potential therapeutic agent for ischemia/reperfusion (I/R)-induced AKI (29, 30). These findings collectively hint at the pharmacological basis of BYHWD’s potential multi-component, multi-target action. However, it is crucial to acknowledge that the efficacy of a TCM formula is not guaranteed by the mere presence of certain components, as the specific formula composition, preparation, and holistic synergy are paramount. Direct evidence for the efficacy of the complete BYHWD formula in AKI and its underlying integrated mechanisms remain largely unexplored.

Given its established role in treating ‘blood stasis’ syndromes and preliminary evidence of its protective effects in ischemic injury in other organs, we hypothesize that BYHWD may also confer protection in AKI. *In vivo* studies initially revealed the renoprotective effect of BYHWD. To decipher the underlying multi-target mechanisms, network pharmacology was employed, constructing a holistic herb-component-target-pathway network consistent with the formula’s polypharmacological nature. The key pathways and targets predicted were then interrogated using BYHWD-containing serum in an *in vitro* model of H/R-injured HUVECs, elucidating its endothelial protective mechanisms.

## 2 Materials and methods

### 2.1 Network pharmacology analysis

To systematically elucidate the potential mechanisms underlying the multi-component, multi-target therapeutic effects of BYHWD on AKI, a predictive study was first conducted using network pharmacology.

#### 2.1.1 Construction of BYHWD active component and target database

To obtain high-quality active components of BYHWD, constituents of its seven constituent herbs were retrieved from the TCMSP (Traditional Chinese Medicine Systems Pharmacology Database) database. Screening criteria were set as oral bioavailability (OB) ≥ 30% and drug-likeness (DL) ≥ 0.18 to ensure favorable pharmacokinetic properties. For components of *Pheretima* (Dilong) not available in TCMSP, the BATMAN-TCM platform was used for supplementation, with a threshold of a corrected confidence score ≥ 80%. All predicted targets were uniformly standardized to official human gene names using the UniProt database, thereby constructing a comprehensive active component-target dataset for BYHWD.

#### 2.1.2 Acquisition of AKI-related targets

AKI-related targets were retrieved from the GeneCards database using the keyword “Acute kidney injury.” The intersection between BYHWD targets and AKI targets was identified using the Venny 2.1.0 tool. These common targets were then imported into Cytoscape software to construct a “BYHWD-Active Components-Common Targets-AKI” network, visually illustrating the potential associations between the drug and the disease.

#### 2.1.3 PPI Network construction

To identify hub proteins within the BYHWD interaction network, the common targets were submitted to the STRING database. A PPI network was constructed with the species limited to “Homo sapiens” and a high confidence interaction score (>0.9). Subsequent topological analysis was performed using the Network Analyzer tool in Cytoscape. Core targets were screened based on network parameters, specifically selecting nodes with a degree value not less than twice the median, for further in-depth investigation.

#### 2.1.4 GO and KEGG pathway enrichment analysis

To interpret the biological functions of the core targets, GO functional enrichment and KEGG pathway enrichment analyses were performed on the common targets using the Metascape platform. A significance threshold of *P* < 0.01 was set, and the top 15 significantly enriched terms in each category were extracted to identify the key biological processes and signaling pathways potentially involved in BYHWD’s action.

#### 2.1.5 Molecular docking validation

To validate the binding potential between active components and core targets, molecular docking was performed. Crystal structures of the core target proteins were obtained from the Protein Data Bank (PDB). The screened active components and core targets were docked using AutoDock Vina. Binding stability was assessed based on the calculated binding energy, and the docking results were visualized using PyMOL.

### 2.2 Preparation of drug-containing serum

To obtain BYHWD-containing serum for *in vitro* experiments, the decoction was prepared according to pharmacopeia standards. The equivalent dose for Sprague-Dawley (SD) rats was calculated based on body surface area conversion. Rats were administered the decoction via gavage for 5 consecutive days. After the last administration, blood was collected aseptically from the abdominal aorta. Serum was separated, heat-inactivated, filter-sterilized, aliquoted, and stored at −20°C.

### 2.3 *In vivo* experiments

#### 2.3.1 Animals and experimental groups

Specific pathogen-free male C57BL/6 mice, aged 6–8 weeks and weighing 22–28 g, were procured from Liaong Changsheng Biotechnology and maintained under standard experimental conditions. The animals were randomly assigned to the following subgroups (*n* = 5 per group). Control Group (Con): Received an equivalent volume of saline orally from the outset of the study. Sham Operation Group (Sham): Subjected to sham surgery, excluding renal pedicle clamping, and administered saline 24 h postoperatively. IRI Group: Underwent IRI surgery and received saline 24 hours postoperatively. Low-Dose BYHWD Group (IRI + L): After IRI surgery, received a low dose of BYHWD (18.525 g/kg) 24 hours postoperatively. High-Dose BYHWD Group (IRI + H): Post-IRI surgery, administered a high dose of BYHWD (37.05 g/kg) 24 h postoperatively. All procedures were conducted under standardized conditions, with post-treatment monitoring for distress or adverse effects. Ethical approval was granted under the number 8177032062.

#### 2.3.2 I/R AKI model

The AKI mouse model was induced by IRI. Mice were anesthetized and maintained at 37°C before kidney exposure. The renal pedicles were clamped with microarterial clips for 45 min to induce ischemia, after which the clips were removed to allow reperfusion, and the surgical site was sutured. The sham group underwent identical procedures except for the clamping of renal pedicles. BYHWD was administered at low (18.525 g/kg) and high (37.05 g/kg) doses, calculated based on body surface area conversion coefficients. Serum and renal tissue samples were collected on day 2 post-reperfusion for analysis.

### 2.4 *In vitro* experiments

#### 2.4.1 Cell model and intervention

To simulate IRI in AKI, a H/R model was established using HUVECs. Cells were subjected to 24 h of severe hypoxia (1% O2) in a tri-gas incubator, followed by 2 h of reoxygenation under normoxic conditions. To determine the safe intervention concentration of BYHWD-containing serum, its effects on cell viability were preliminarily assessed using the CCK-8 assay across different serum concentrations.

#### 2.4.2 Experimental groups

Groups: Control: Basal medium (37°C, 5% CO_2_, 24 h); H/R: Hypoxia-reoxygenation treatment; H/R + 10%BS: 2.5% blank serum + H/R; H/R + 2.5%DS: 2.5% medicated serum + H/R; H/R + 10%DS: 10% medicated serum + H/R

#### 2.4.3 Cell viability and apoptosis detection

To evaluate the protective effects of BYHWD against H/R-induced cell injury, cell proliferation viability was measured using the CCK-8 assay, and cell survival rate was calculated via Trypan Blue staining. Furthermore, quantitative analysis of apoptosis levels was performed using Annexin V-FITC/PI double staining combined with flow cytometry.

#### 2.4.4 Oxidative stress level detection

To investigate whether BYHWD exerts its effects by alleviating oxidative stress, intracellular reactive oxygen species (ROS) levels were labeled using the DCFH-DA fluorescent probe and quantified by flow cytometry in different experimental groups.

#### 2.4.5 Western blot analysis

To validate the predictions from network pharmacology at the molecular level and explore the specific mechanisms, the expression and phosphorylation levels of key proteins related to apoptosis (Bax, Bcl-2), endothelial function (CD31, MCP-1, α-SMA), and the predicted core pathways (VEGFRII, PI3K/AKT/FOXO1) were detected by Western blotting.

### 2.5 Statistical analysis

All experiments were independently repeated at least three times. Data are presented as mean ± standard deviation. Statistical analysis was performed using GraphPad Prism 8 software. Comparisons among multiple groups were analyzed by one-way analysis of variance (ANOVA) followed by Tukey’s *post-hoc* test. A *P* < 0.05 was considered statistically significant.

### 2.6 Reagents

The following reagents were used: (HUVEC)—-ATCC; (Fetal Bovine Serum, FBS) -(Thermo Fisher Scientific); (Incomplete RPMI—-1640 Medium without double antibodies)—-(KGI Biotechnology-KGL1503-500); (CCK8 working solution)—-(KGI Biotechnology-KGA9310-500); (Reactive Oxygen Species Assay Kit)—-S0033M- (Beyotime Biotechnology); Anti-beta Actin Antibody—-(Abcam-ab-6276); Anti-α-SMA Antibody—-(Abcam-ab5694); Anti-Bcl-2 Antibody—-(Abcam-ab59348); Anti-CD31 Antibody—-(Cell Signaling Technology, CST-CST-3528); Anti-Bax Antibody—-(Santa Cruz Biotechnology-sc7480); Anti-FUT8 Antibody—-(Cohesion Biosciences-sc68941); Anti-VEGFR2 Antibody—-(Cohesion Biosciences-sc-393163); Anti-TGFβR2 Antibody—-(Abcam-ab61213); Anti-MCP-1 Antibody—-(Cell Signaling Technology, CST-25542-1); Phospho-AKT Antibody -(Cell Signaling Technology, CST-9271-T); Phospho-PI3Kinasep85 Antibody—-(Cell Signaling Technology, CST-4228-T); Anti-PI3Kinasep85 Antibody—-(Cell Signaling Technology, CST-4292-S); Phospho-FoxO1 Antibody—-(Cell Signaling Technology, CST-9461-T); Anti-FoxO1 Antibody -(Cell Signaling Technology, CST-2880-T).

## 3 Results

### 3.1 BYHWD ameliorated the renal impairment detected in AKI model mice

**FIGURE 1:**
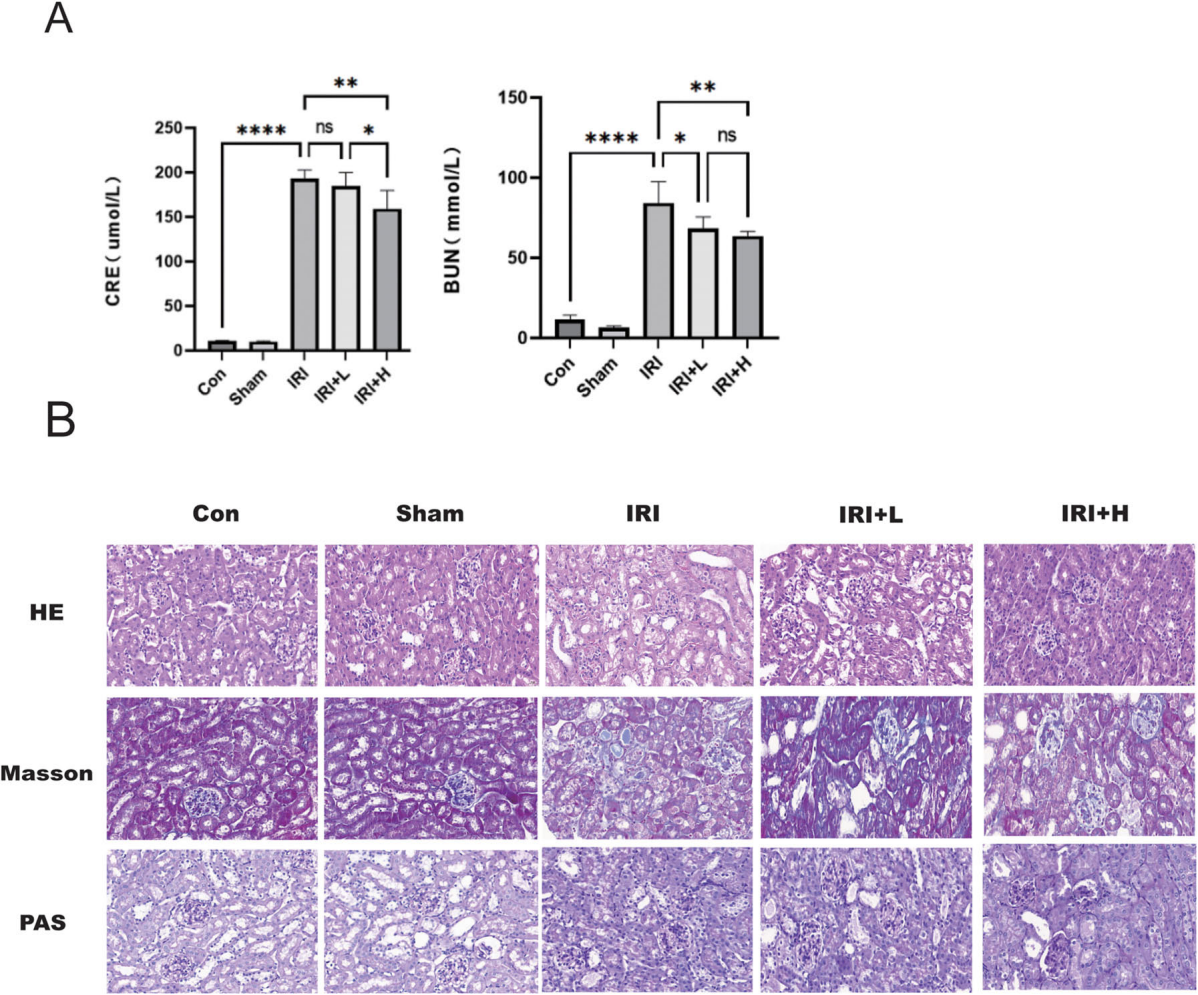
**(A)** Serum CRE and BUN levels of renal IRI model mice 2 days after treatment with BYHWD. Significant differences are indicated by asterisks (*p < 0.05, **p < 0.01, ****p < 0.0001). **(B)** HE, Masson, PAS staining of renal tissue from IRI model mice at 2 days after treatment with BYHWD. *Con*: Control group; Sham: Sham operation group; *IRI*: Ischemia-reperfusion group; 1. HE staining: Reveals tubular epithelial cell vacuolar degeneration (black arrows) and pyknosis/karyorrhexis (indicating necrosis). Renal interstitial edema with inflammatory cell infiltration is also present (asterisks). 2. Masson’s staining: Demonstrates widened interstitium with collagen fiber/extracellular matrix deposition (blue arrows), representing an acute interstitial response to injury. 3.PAS staining: Shows loss of the brush border and glycogen in tubular epithelial cells (triangles), along with disruption and discontinuity of the tubular basement membrane (red arrows). IRI + L: BYHWD low-dose group; IRI + H: BYHWD high-dose group; 1. HE staining: Compared with the IRI group, the treatment groups exhibit a significant reduction in tubular epithelial cell vacuolar degeneration and necrosis, along with markedly attenuated interstitial edema and inflammation. 2. Masson’s trichrome staining: Compared with the IRI group, the treatment groups show decreased collagen/matrix deposition in the interstitium, indicating amelioration of the acute interstitial response. 3. PAS staining: Compared with the IRI group, the treatment groups demonstrate partial restoration of the brush border and glycogen content in tubular epithelial cells, as well as improved continuity of the tubular basement membrane (red arrows).

The serum CRE and BUN levels of renal IRI model mice detected at day 2 (during the AKI phase) after treatment with and without BYHWD are shown in Table 1. Compared with the control group, significantly elevated levels of serum CRE and BUN were detected in the IRI group (both *P* < 0.0001). Notably, compared with the levels in the IRI group, the serum levels of CRE were reduced in both the IRI-L and IRI-H groups (*P* > 0. 05 and *P* < 0. 01, respectively). Compared with the levels in the IRI-L group, the serum levels of CRE were reduced in IRI-H group (*P* < 0.05). The results were dose-dependent. The levels of BUN were also reduced in both the IRI + L and the IRI + H groups compared with those detected in the IRI group (*P* < 0.05 and *P* < 0.01, respectively). Compared with the levels in the IRI-L group, the serum levels of BUN were reduced in IRI-H group (*P* > 0.05) (Figure 1A). The results were dose-dependent. These data indicate that BYHWD treatment could improve renal function.

**TABLE 1:** Serum CRE and BUN levels of renal IRI model mice 2 days after treatment with BYHWD.

Group	CRE (μmol/L)	BUN (mmol/L)
Con	10.8 ± 0.447	11.252 ± 2.897
Sham	10 ± 0.707	6.614 ± 0.959
IRI	191.2 ± 9.203	86.26 ± 12.458
IRI + L	180.2 ± 16.574	68.702 ± 6.093
IRI + H	156.8 ± 18.512	64.172 ± 2.517

Con, Control group; Sham, Sham operation group; IRI, Ischemia-reperfusion group; IRI + L, BYHWD low-dose group; IRI + H, BYHWD high-dose group.

### 3.2 BYHWD reduced inflammatory cell infiltration of renal tissue detected in AKI model mice

Renal tissue samples collected at 2 days post-modeling were stained with HE to assess the level of inflammatory cell infiltration during the AKI phase (Figure 1B). Compared with control group, more inflammatory cell infiltration was observed in the renal tissue of IRI model mice. Notably, reduced inflammatory cell infiltration was observed in the renal tissue of IRI model mice at 2 days after treatment with BYHWD in both the IRI + L and IRI + H groups. These data indicate that BYHWD treatment reduces inflammatory cell infiltration associated with kidney injury.

### 3.3 Screening of active components in BYHWT

Using pharmacokinetic screening criteria [OB ≥ 30% and drug-likeness (DL) ≥ 0.18], active components of BYHWD were systematically retrieved from the TCMSP database. Screening results revealed the following constituent counts: Huangqi (20 active components), Danggui (3), Chishao (29), Chuanxiong (7), Honghua (22), and Taoren (23). As components of Dilong were unavailable in TCMSP, 30 components from Dilong were supplemented via the BATMAN-TCM database, with subsequent exclusion of 10 components due to unavailable target information. To standardize the target database, UniProt database was employed for gene name normalization of target proteins. Following data integration and deduplication, 444 non-redundant therapeutic targets of BYHWD were identified. Target contributions per herb were quantified as: Huangqi (186 targets), Chishao (84), Danggui (46), Chuanxiong (24), Dilong (268 targets), Honghua Flos (184), and Taoren (40). This standardized target database establishes a robust foundation for subsequent network pharmacology analyses.

### 3.4 Construction of AKI disease targets and herb-component-target network

**FIGURE 2:**
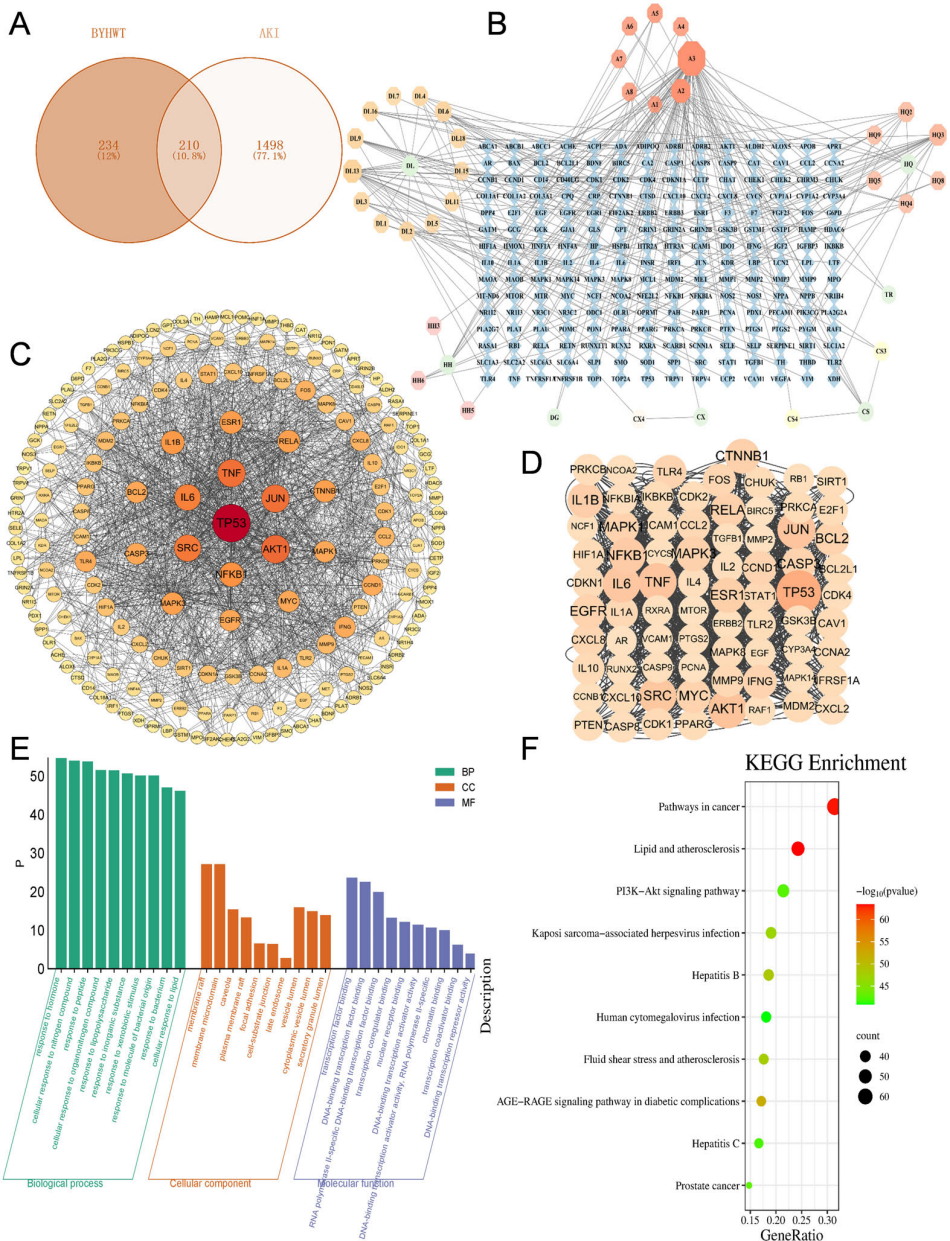
**(A)** Venn diagram of traditional Chinese medicine-related disease actions. **(B)** Network diagram of drug-ingredient-target interaction. Green circles represent TCM (7 in total); Blue rhombuses represent targets (210 in total); Orange hexagons represent overlapping active ingredients of TCM (8 in total A1, Huangqi, Taoren; A2, Huangqi, Honghua; A3, Huangqi, Honghua; A4, Danggui, Chishao, Honghua, Taoren; A5, Danggui, Chishao, Honghua; A6, Chishao, Honghua; A7, Chishao, Honghua; A8, Chishao, Chuanxiong). The larger the shape, the more genes targeted by the active ingredient. **(C)** PPI Network and Key Targets of BYHWD Treatment on AKI. In the visualization representation of protein-protein interaction (PPI) networks, circular topological units represent target genes. The geometric size and chromatic gradient of nodes are positively correlated with their topological importance (quantitatively expressed through degree centrality, betweenness centrality, etc.). Edge attributes are characterized by chromatic differences to represent interaction types and strength, where darker lines indicate strong associations that are experimentally validated or statistically significant. **(D)** Identification of core therapeutic targets for BYHWD in treating AKI through PPI network analysis. **(E)** GO Enrichment Analysis of Potential Targets for BYHWD in Treating AKI. This study visualizes the Gene Ontology enrichment results using a three-category bar chart. The horizontal axis displays the top 10 significantly enriched pathway names in three categories: Biological Process (BP), Cellular Component (CC), and Molecular Function (MF). The vertical axis uses a -log10 (*P*-value) scale, which linearizes the representation of statistical significance levels; higher values indicate more significant enrichment. The height of the bars in the chart is positively correlated with the significance level, and the color gradient of the bars reflects the original *P*-value size (darker colors indicate smaller *P*-values). A larger *P*-value indicates a higher number of genes involved, suggesting higher enrichment. **(F)** KEGG Enrichment Analysis of Potential Targets for BYHWD Treatment of AKI. The bubble chart visually presents enrichment characteristics through multiple dimensions: The vertical axis (Y-axis) labels KEGG pathway entries, while the horizontal axis (X-axis) represents Gene Ratio, which is the ratio of enriched gene numbers to the total differential gene numbers. A higher ratio indicates a higher degree of pathway enrichment. The color of the bubbles adopts a green-to-red gradient scale, reflecting the significance level after multiple testing correction (*p*-value). Colors approaching red indicate smaller *p*-values and more statistically significant enrichment.

A systematic screening of the GeneCards database identified 8,826 AKI-related disease genes. By applying a gene-disease relevance score threshold (score ≥ 10), 1,708 high-relevance candidate genes were selected. Subsequent intersection with BYHWD’s active component targets yielded 210 shared drug-disease targets (Figure 2A), suggesting potential therapeutic loci for BYHWD in AKI treatment. To elucidate the molecular mechanisms underlying multi-component synergy, a three-dimensional topological network model integrating BYHWD’s active components, therapeutic targets, and AKI disease networks was constructed using Cytoscape 3.10.1 (Figure 2B). This visualization elucidated multi-level interaction relationships between herbal components and disease targets within the TCM formulation. Node degree centrality quantified regulatory weights of individual targets, while spatial distribution patterns of hub targets suggested their potential roles as network hubs mediating critical biological pathways.

### 3.5 PPI network analysis

A systems biology approach was employed to construct the protein interaction network. Firstly, the intersection dataset comprising 210 candidate targets was submitted to the STRING database (v12.0), where a multi-evidence integration algorithm generated a PPI network. To ensure reliability, a confidence threshold > 0.900 (high confidence) was applied, representing a probability value calculated from experimental validation, homology inference, co-expression, and other evidence. Following topological optimization, 18 isolated nodes lacking interaction associations were removed, yielding a refined network of 192 core targets. Data were exported in tab-separated values (TSV) format and imported into Cytoscape 3.10.1 for visualization and modular analysis. A force-directed layout algorithm optimized spatial architecture, while the MCODE plugin identified functional clusters. As shown in Figure 2C, the PPI network comprised 190 nodes and 1,472 interaction edges. Further calculations revealed a median node degree of 7.02. Nodes with degree values ≥ 14.04 (twice the median) were defined as critical targets, ultimately identifying 76 BYHWT-associated therapeutic targets for AKI (Figure 2D). Additionally, a table of the top 20 bioactive components was generated based on topological metrics (degree, betweenness, closeness centrality) (Table 2).

**TABLE 2:** Topological parameters of network nodes for the top 20 active components.

Targets name	Degree	Betweenness centrality	Closeness centrality
TP53	108	0.198314514	0.419068736
AKT1	68	0.083512479	0.421875000
TNF	66	0.097528664	0.420000000
JUN	66	0.102272403	0.431506849
SRC	62	0.151150136	0.415384615
IL6	60	0.062466181	0.397058824
NFKB1	52	0.040741967	0.402127660
IL1B	48	0.019178535	0.377245509
ESR1	46	0.028912275	0.403846154
RELA	44	0.020684528	0.392116183
CTNNB1	44	0.037981131	0.397894737
MAPK3	44	0.046849217	0.406451613
BCL2	40	0.034047431	0.362068966
CASP3	40	0.034869580	0.386503067
EGFR	40	0.042100538	0.398734177
MAPK1	40	0.016284098	0.400423729
MYC	40	0.012712357	0.372781065
CCND1	38	0.008347445	0.363461538
TLR4	38	0.047975707	0.362763916
IFNG	38	0.045451433	0.357277883

### 3.6 GO and KEGG pathway analyses of BYHWD in AKI treatment

GO functional and KEGG pathway enrichment analyses were conducted via the Metascape platform using a significance threshold of *P* < 0.01. The GO analysis encompassed three categories: biological processes (BP), cellular components (CC), and molecular functions (MF), with the top ten enriched pathways from each category selected for visualization. Bar charts illustrating these results were generated using the bioinformatics tool Microbioinformatics (Figure 2E).

Systematic biological analysis of raw data revealed potential mechanisms underlying BYHWD’s therapeutic effects on AKI through KEGG pathway enrichment. After applying a significance threshold (*P* < 0.05, FDR-corrected), 219 significantly enriched signaling pathways were identified. The top 10 most statistically significant pathways (ranked by ascending *P*-values) were categorized into three functional clusters (Table 3). A bubble plot (Figure 2F), generated via the Microbioinformatics online platform,1 comprehensively visualized the enrichment results. Notably, the PI3K-AKT signaling pathway emerged as a potential central regulator. This pathway is critically involved in promoting cell survival, inhibiting apoptosis, and modulating cellular metabolism. In the context of AKI, its significant enrichment (enrichment factor: 0.29) suggests that BYHWD may alleviate renal tubular epithelial cell injury primarily by activating these pro-survival and anti-apoptotic mechanisms. The identification of 18 differential genes (12.3% of total pathway genes) further supports a substantial impact of BYHWD on this pathway. Moreover, the substantial overlap with apoptosis-regulating pathways indicates a coordinated regulatory network: PI3K-AKT signaling likely crosstalks with and suppresses the FoxO pathway and modulates p53-mediated apoptotic responses. This integrated action would collectively enhance the resilience of renal tubular cells to the ischemic or toxic insults characteristic of AKI, positioning the PI3K-AKT axis as a key therapeutic hub for BYHWD.

**TABLE 3:** Ten highly significant pathways in BYHWD intervention for AKI.

Pathway category	Pathway name	KEGG ID
Metabolism-related pathways	Lipid metabolism and atherosclerosis	hsa05417
	AGE-RAGE signaling pathway in diabetic complications	hsa04933
	Fluid shear stress and atherosclerosis	hsa05418
Malignant proliferation-related pathways	Protein kinase signaling transduction in cancer;	hsa05200
	Prostate cancer	hsa05215
	PI3K-AKT signaling pathway	hsa04151
Pathogen-host interaction pathways	Hepatitis B	hsa05161
	Hepatitis C	hsa05160
	Kaposi’s sarcoma-associated herpesvirus infection	hsa05167
	Human cytomegalovirus infection	hsa05163

### 3.7 Molecular docking validation and visualization

**FIGURE 3:**
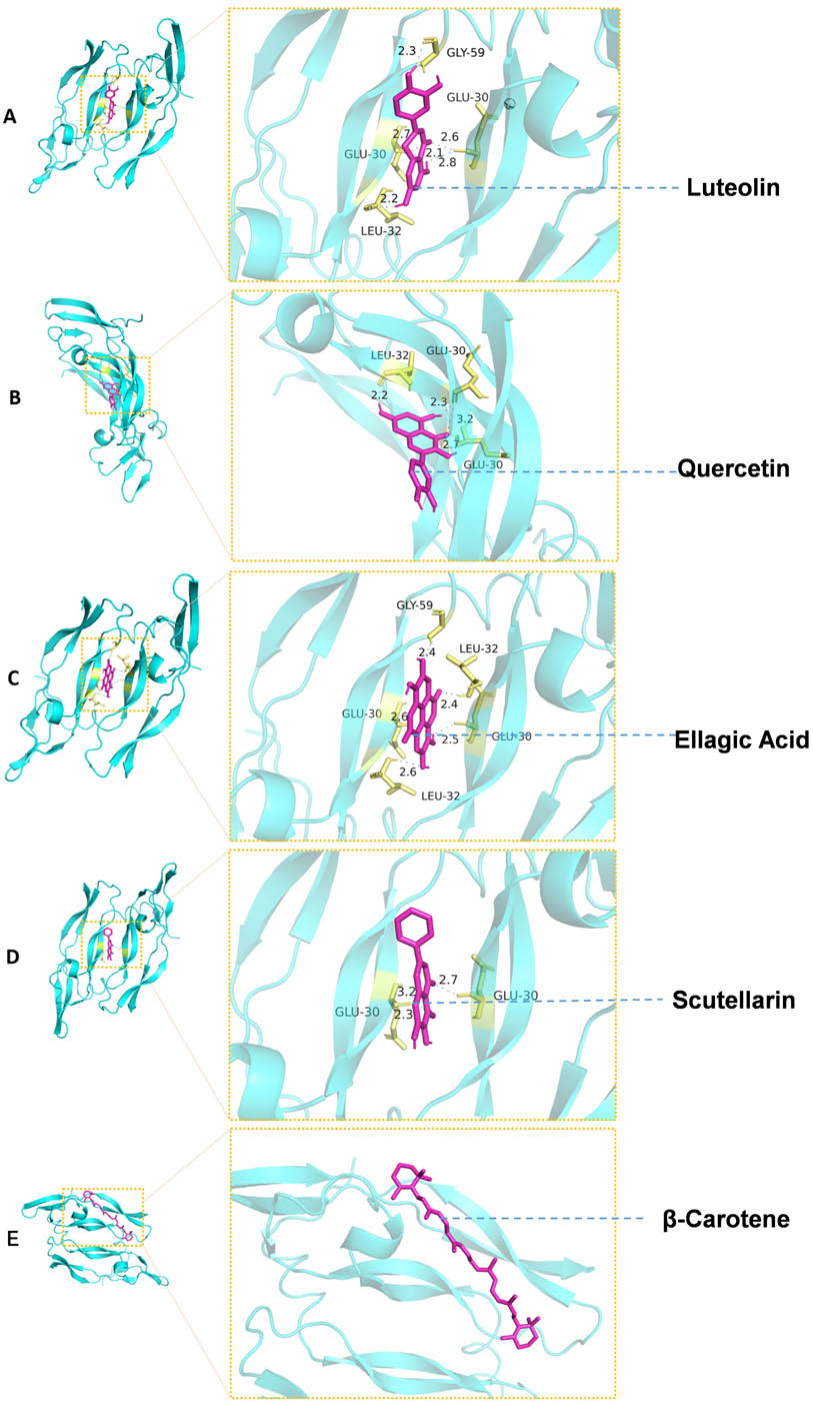
Molecular docking visualization. **(A–E)** represent the molecular docking results of luteolin, quercetin, ellagic acid, baicalin, β-carotene, and VEGFA, respectively. Among them, the molecular docking visualization of β-carotene and VEGFA shows no hydrogen bonds, which may be due to the large hydrophobic region of β-carotene. This could potentially form hydrophobic interactions with the hydrophobic part of VEGFA, thereby enhancing the binding energy.

Receptor proteins (PDBQT format) and ligand molecules (PDBQT format) were loaded into AutoDock Vina, and the docking program was executed. During computation, the software automatically optimized the ligand’s spatial orientation and conformational geometry to identify the optimal binding mode with the receptor. Following docking completion, ligand-receptor binding conformations were generated, and free binding energy values were calculated (Table 4). More negative free binding energy values indicate greater stability of ligand-receptor interactions. Finally, PyMOL software was employed to visualize the docking results, illustrating the molecular interactions between ligands and receptors (Figure 3).

**TABLE 4:** Generation of binding free energy between ligand molecules and receptor proteins.

Molecule name	Mol ID	Source	Binding energy
Ellagic Acid	MOL001002	Chishao	−4.42
Quercetin	MOL000098	Huangqi	−4.73
Scutellarin	MOL002714	Chishao	−4.82
β-Carotene	MOL002773	Honghua	−5.42
Luteolin	MOL000006	Honghua	−6.07

The more negative the binding energy, the more stable the binding between the ligand and the receptor.

### 3.8 Effects of BYHWD drug-containing serum at different concentrations on HUVEC proliferation

**FIGURE 4:**
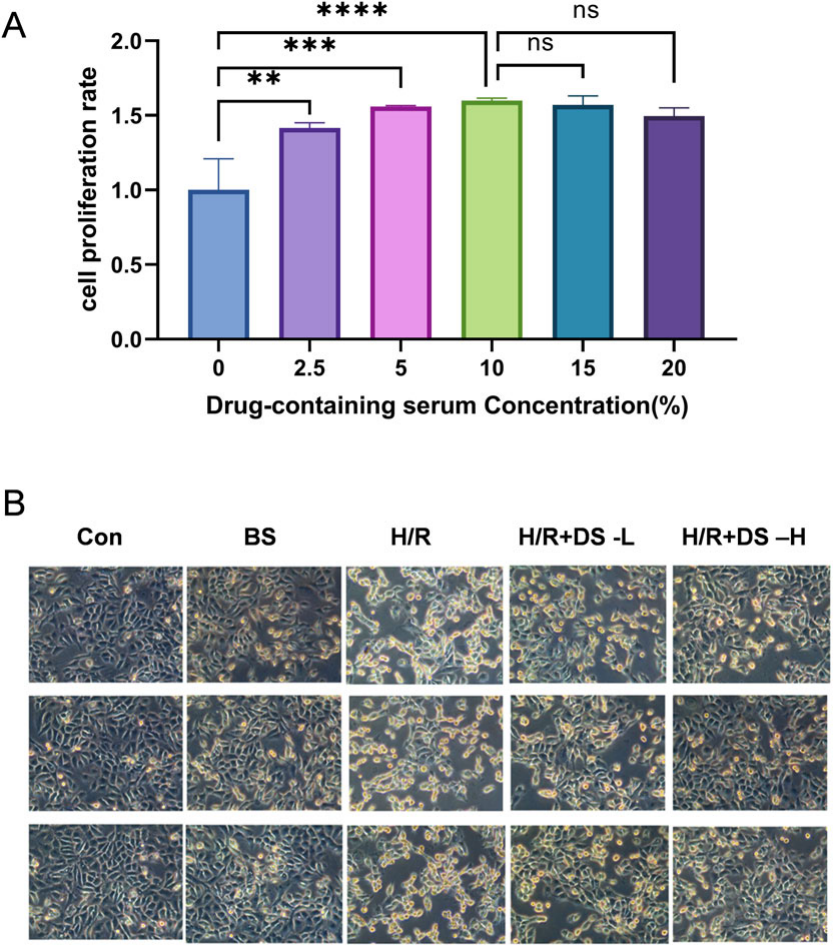
**(A)** Changes in the survival rate of HUVECs after 24 h of stimulation with serum containing different drug concentrations. **(B)** Trypan blue staining of HUVEC cells under light microscopy (×200). Significant differences are indicated by asterisks (**p < 0.01, ***p < 0.001, ****p < 0.0001).

To evaluate the dose-response relationship of BYHWD drug-containing serum on vascular endothelial cell proliferation, the CCK-8 assay was employed to measure viability changes in HUVECs after 24-h treatment with serum at varying concentrations (0, 2.5, 5, 10, 15, 20%). Experimental groups included a blank control (0%” serum group in the cell viability assay refers to the group where no drug-containing serum was added, meaning it was supplemented with 10% blank control serum (10% BS)) and five concentration gradients, with six technical replicates per concentration. Cell culture conditions were maintained at 37°C with 5% CO2. As shown in Figure 4A, the drug-containing serum significantly enhanced proliferation at concentrations of 2.5–10% (*P* < 0.01, one-way ANOVA). Notably, a plateau phase in proliferation rate was observed at concentrations exceeding 10%, with no statistically significant differences in cell viability between the 15 and 20% groups compared to the 10% group (*P* > 0.05). Based on this dose-response curve, subsequent experiments selected 2.5% as the low-concentration intervention group and 10% as the high-concentration group.

### 3.9 Effects of BYHWD drug-containing serum on HUVEC survival rate under H/R injury

To evaluate the effect of BYHWD drug-containing serum on the survival rate of HUVECs after H/R injury, this study quantified cell viability using the trypan blue staining method. As shown in Figure 4B, following H/R treatment, cells in each experimental group were stained with 0.4% trypan blue solution (final concentration 0.04%) for 3 min. Morphological changes and staining characteristics were observed under an optical microscope (200× magnification). Morphological analysis revealed that cells in the Control group exhibited the typical morphology of endothelial cells, characterized by a regular polygonal structure, intact cell membranes, and tight adherence. In contrast, the H/R group displayed significant pathological alterations: the majority of cells showed extensive blue staining, abnormal cellular swelling, blurred boundaries, and detachment, indicating severe membrane structural damage. Notably, intervention with 2.5% and 10% BYHWD drug-containing serum significantly ameliorated H/R-induced cellular injury. Cells maintained an intact spindle-shaped structure with higher density of adherent cells.

### 3.10 BYHWD drug-containing serum attenuates H/R-induced apoptosis in HUVECs

**FIGURE 5:**
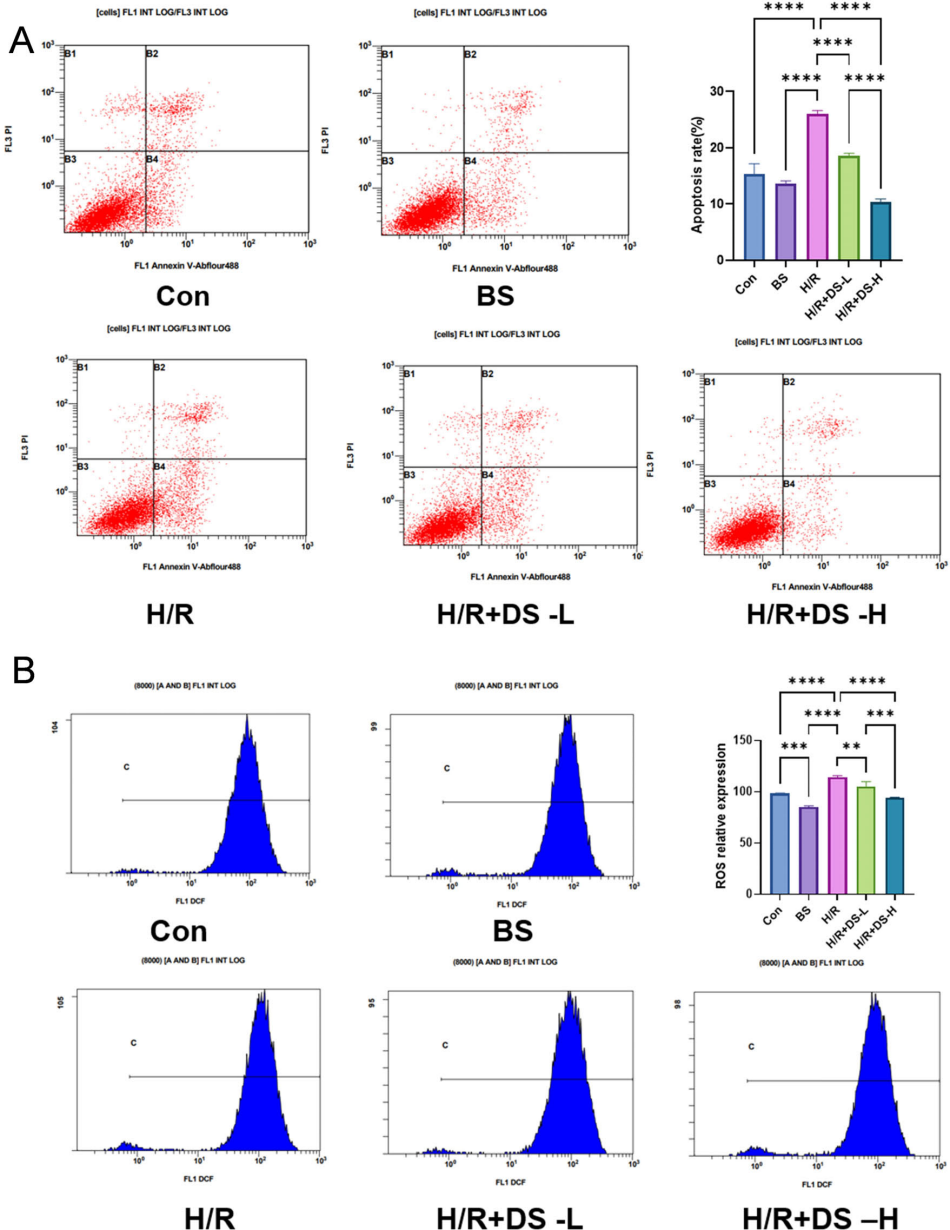
**A**: Detection of apoptosis in HUVEC groups using Annexin V/PI dual staining combined with flow cytometry. **B**: Flow cytometry analysis of oxidative stress levels in HUVEC cells. Significant differences are indicated by asterisks (**p < 0.01, ***p < 0.001, ****p < 0.0001).

To systematically evaluate the effects of BYHWD drug-containing serum on H/R-induced apoptosis in HUVECs, quantitative analysis was performed using Annexin V-FITC/PI dual staining coupled with flow cytometry (Figure 5A). Results demonstrated that the total apoptosis rate in the H/R model group significantly increased to (26.1 ± 0.6) % compared to the normal control group (*P* < 0.01), confirming successful establishment of the endothelial apoptosis model. In intervention groups, treatment with 2.5 and 10% drug-containing serum reduced apoptosis rates to (18.5 ± 0.5) % and (10.3 ± 0.68%, respectively (both *P* < 0.01 vs. H/R group).

### 3.11 Regulatory effects of BYHWD drug-containing serum on oxidative stress in HUVECs following H/R injury

To evaluate oxidative stress responses in endothelial cells under H/R injury and the therapeutic effects of drug intervention, intracellular ROS levels were measured via flow cytometry. As shown in Figure 5B, the rightward shift of fluorescence signals correlated positively with ROS levels. Experimental results demonstrated a significant increase in ROS fluorescence intensity in the H/R model group compared to the normal control group (*P* < 0.01, one-way ANOVA), indicating elevated oxidative stress. Treatment with 2.5 and 10% BYHWD drug-containing serum significantly reduced ROS production compared to the model group (both *P* < 0.01), suggesting that BYHWD effectively mitigates H/R-induced oxidative damage in endothelial cells.

### 3.12 BYHWD reduced the expression of NGAL detected in renal tissue of AKI model mice

**FIGURE 6:**
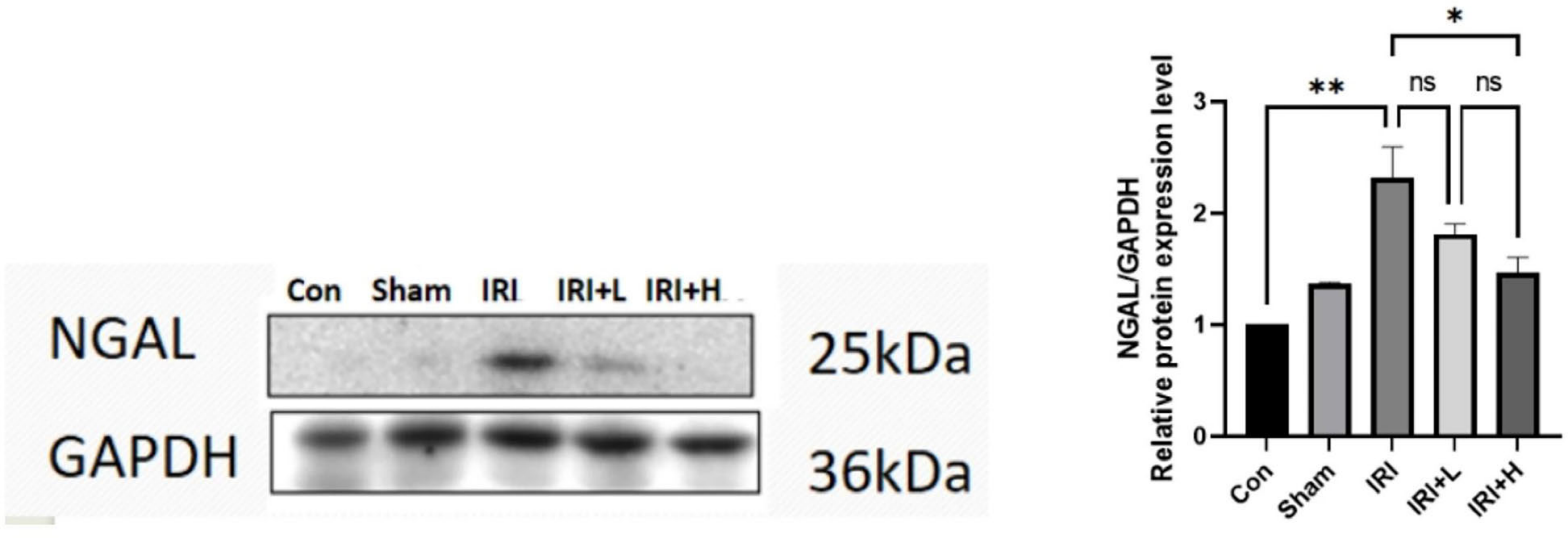
Western blot analysis of NGAL expression in renal tissue from IRI model mice at 2 days after treatment with BYHWD. Con, Control group; Sham, Sham operation group; IRI, Ischemia-reperfusion group; IRI + L, BYHWD low-dose group; IRI + H, BYHWD high-dose group. Significant differences are indicated by asterisks (*p < 0.05, **p < 0.01).

We also detected the expression levels of NGAL as a biomarker of AKI in the renal tissue of IRI model mice at day 2 after treatment with and without BYHWD. As shown in Figure 6, Western blot analysis revealed significantly elevated expression of NGAL in renal tissue of the IRI group compared with that in the control group (*P* < 0.01). In contrast, the expression of NGAL was expression was reduced in renal tissue in the IRI + L and IRI + H groups compared with that in the control group (*P* > 0. 05, *P* < 0.05). Compared with the levels in the IRI-L group, the expression level of NGAL was reduced in IRI-H group (*P* > 0.05). The results were dose-dependent. These data indicate that BYHWD treatment reduces inflammatory factors associated with kidney injury.

### 3.13 Regulatory effects of BYHWD drug-containing serum on H/R-induced endothelial cell apoptosis

**FIGURE 7:**
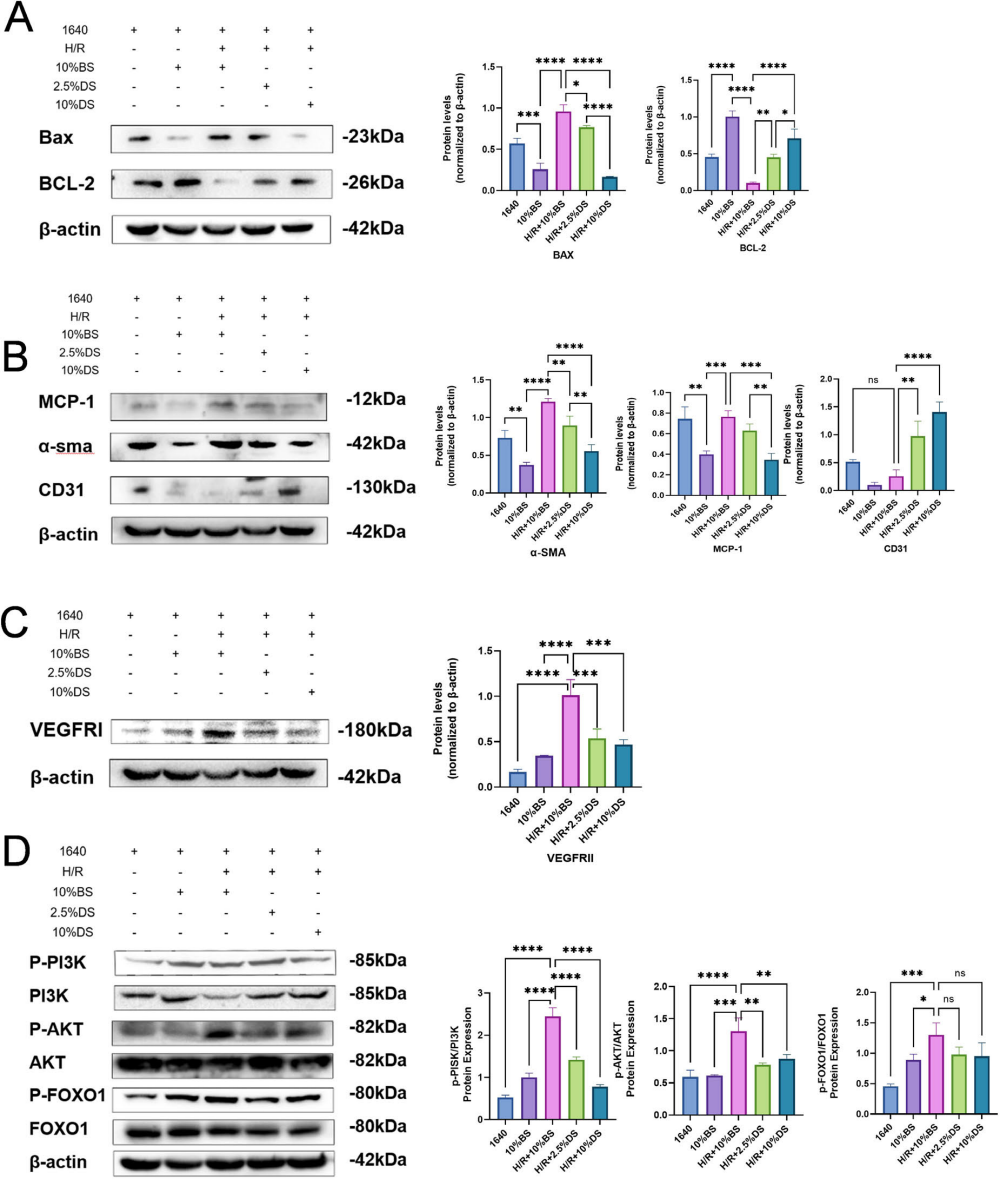
**(A)** Changes in the expression of BAX and BCL-2 proteins in various cell groups. **(B)** Western blot analysis of MCP-1, CD-31, and α-SMA protein expression changes in each group of cells. **(C)** Western blot analysis of VEGFRII protein expression in cells from each group. **(D)** Changes in protein expression of p-PI3K, PI3K, p-AKT, AKT, p-FOXO1, and FOXO1 in each group of cells. Significant differences are indicated by asterisks (*p < 0.05, **p < 0.01, ***p < 0.001, ****p < 0.0001).

To systematically evaluate the anti-apoptotic mechanisms of BYHWD drug-containing serum, quantitative analysis of apoptosis-related protein expression profiles in HUVECs under H/R injury was performed using Western blotting. As shown in Figure 7A, compared to the normal culture group (Control), the H/R model group exhibited significant upregulation of the pro-apoptotic protein Bax (*P* < 0.01) and downregulation of the anti-apoptotic protein Bcl-2 (*P* < 0.01). Notably, intervention with BYHWD drug-containing serum reduced Bax expression (*P* < 0.01 vs. H/R group) while elevating Bcl-2 levels (*P* < 0.01). These data confirm that BYHWD drug-containing serum significantly attenuates H/R-induced cellular damage by modulating the balance of Bcl-2 family protein expression, suggesting its critical role in regulating the mitochondrial-dependent apoptotic pathway.

### 3.14 Regulatory effects of BYHWD-containing serum on endothelial cell mesenchymal transition and inflammatory markers

To investigate the inflammatory response and mesenchymal transition characteristics of endothelial cells under H/R injury and evaluate the protective effects of BYHWD-containing serum, this study analyzed the protein expression levels of monocyte chemoattractant protein-1 (MCP-1), α-smooth muscle actin (α-SMA), and CD31 in HUVECs. Experimental results (Figure 7B) showed that compared with the control group, H/R injury significantly upregulated the expression of MCP-1 (*P* < 0.01, *P* < 0.01) and α-SMA (*P* < 0.01, *P* < 0.01), while suppressing CD31 expression (*P* < 0.01, *P* < 0.01). After intervention with BYHWD-containing serum, both the H/R + 2.5% DS (drug-containing serum) and H/R + 10% DS groups exhibited dose-dependent regulatory effects: MCP-1 and α-SMA expression levels were significantly reduced compared to the H/R group (*P* < 0.01, *P* < 0.01), and CD31 expression was markedly restored (*P* < 0.01, *P* < 0.01). These findings suggest that BYHWD effectively inhibits the mesenchymal transition process in HUVECs and mitigates H/R-induced inflammatory responses.

### 3.15 Regulatory effect of BYHWD drug-containing serum on the VEGF/VEGFRII pathway

To investigate the effect of BYHWD drug-containing serum on the vascular endothelial growth factor receptor II (VEGFRII) signaling pathway, we established a H/R injury model using HUVECs. The protein expression levels of VEGFRII in each group were detected by Western blot, and the results are shown in Figure 7C. In the experimental design, cells were divided into three groups: normal control group (Control), H/R model group (H/R), and H/R model combined with different concentrations of BYHWD drug-containing serum treatment group (H/R + DS). Quantitative analysis revealed that compared to the Control group, the protein expression of VEGFRII in the H/R group was significantly increased (*P* < 0.01, *P* < 0.01), suggesting that H/R stimulation activates the VEGF/VEGFRII signaling pathway. After intervention with BYHWD drug-containing serum, the expression of VEGFRII in the H/R + DS group was inhibited, showing a statistically significant difference (*P* < 0.01, *P* < 0.01). These results indicate that BYHWD drug-containing serum may regulate the activity of the VEGF/VEGFRII signaling pathway by suppressing the overexpression of VEGFRII.

### 3.16 Regulatory effect of BYHWD-medicated Serum on the PI3K/AKT/FOXO1 pathway

To investigate the impact of BYHWD-medicated serum on the PI3K/AKT/FOXO1 signaling pathway in the H/R-injured model, this study employed Western blot to quantitatively analyze the phosphorylation levels of key proteins. The results (Figure 7D) demonstrated that compared with the normal control group, the H/R model group exhibited significantly reduced ratios of p-PI3K/PI3K, p-AKT/AKT, and p-FOXO1/FOXO1 (*P* < 0.05). However, after intervention with 2.5 and 10% BYHWD-medicated serum, the aforementioned phosphorylation ratios showed a statistically significant increase compared to the H/R + 2.5% blank serum group (*P* < 0.05). This suggests that BYHWD may exert protective effects by activating the PI3K/AKT/FOXO1 pathway.

## 4 Discussion

### 4.1 Association of core targets with AKI

The core targets identified in this study—TP53, AKT1, VEGFA, and IL6—play critical roles in the pathogenesis of AKI, involving inflammatory responses, apoptosis, angiogenesis, and signaling pathway regulation.

TP53 (tumor protein 53), a key tumor suppressor gene, influences the progression and repair of renal injury in AKI by regulating apoptosis and autophagy. Studies have demonstrated that TP53 activation promotes the repair of damaged renal tubular epithelial cells. However, excessive p53 activation may exacerbate renal injury by increasing apoptosis. Additionally, TP53 modulates inflammatory responses and oxidative stress, further impacting AKI pathogenesis (31–34).

AKT1, a serine/threonine protein kinase, is involved in cell survival, proliferation, and metabolic regulation. In AKI, AKT1 promotes the survival and repair of renal tubular epithelial cells by activating the PI3K/AKT/mTOR signaling pathway (35, 36). Research indicates that AKT1 activation alleviates IRI-induced renal tubular necrosis, thereby protecting renal function (37). However, excessive activation of AKT1 may also contribute to fibrosis, indicating its dual role in AKI pathogenesis (38).

VEGFA, a pivotal regulator of angiogenesis, primarily exerts protective effects in AKI by promoting vascular regeneration and repairing damaged tissues (39). VEGFA enhances glomerular filtration, improves renal hemodynamics, and facilitates the regeneration of renal tubular epithelial cells via activation of the VEGF receptor (VEGFR) signaling pathway (40, 41). Furthermore, VEGFA exhibits anti-inflammatory and anti-fibrotic properties, mitigating renal damage following AKI (39).

Interleukin-6 (IL-6), a pleiotropic pro- and anti-inflammatory cytokine, is central to AKI pathogenesis (42). IL-6 binds to its receptor (IL-6R) and glycoprotein 130 (gp130), activating the Janus kinase/signal transducer and activator of transcription (JAK-STAT) signaling pathway. This process induces inflammatory responses, exacerbates renal tubular epithelial cell injury, and promotes fibrosis (43). In sepsis-associated AKI, elevated IL-6 levels correlate strongly with the deterioration of renal function (44). Moreover, IL-6 amplifies renal inflammation by modulating immune cell activity (45).

### 4.2 Mechanisms of key signaling pathways

KEGG enrichment analysis revealed that the PI3K-AKT and AGE-RAGE signaling pathways are central to the therapeutic effects of BYHWD on AKI.

The PI3K/AKT signaling pathway is a critical intracellular signaling system involved in regulating diverse cellular activities. AKT, a serine/threonine kinase, phosphorylates multiple substrates to modulate cellular proliferation, differentiation, apoptosis, and migration. This pathway plays a significant role in oxidative stress-induced injury and fibrosis across multiple organs (46, 47). Studies indicate that the PI3K/AKT pathway exerts multiple biological effects by regulating downstream key molecules. For instance, activation of the PI3K/AKT/mTOR pathway upregulates VEGF expression, thereby promoting cerebral angiogenesis (48). Additionally, activation of the PI3K/AKT/eNOS pathway inhibits oxidized low-density lipoprotein (ox-LDL)-induced inflammatory responses and endothelial dysfunction in mouse aortic endothelial cells (49).

The AGE-RAGE pathway, extensively implicated in diabetic nephropathy (50), not only provides a complex interface between inflammation, oxidative stress, and immune responses but also serves as an endothelial adhesion receptor for leukocyte integrins, facilitating the recruitment and extravasation of infiltrating cells. Furthermore, AGE-RAGE interactions induce VEGF production via NF-κB and AP-1 transcription factors, triggering angiogenesis (51).

### 4.3 Synergistic effects of active components

Based on the network pharmacology analysis, this study further validated the key target VEGFA in the PI3K-AKT signaling pathway through molecular docking. Results demonstrated that ellagic acid, quercetin, baicalein, luteolin, and β-carotene from BYHWD exhibited binding affinity with the VEGFA protein (binding energy ranging from −4.73 to −6.07 kcal/mol). Notably, luteolin (−6.07 kcal/mol) and β-carotene (−5.42 kcal/mol) demonstrated higher binding affinity, suggesting that BYHWD may exert therapeutic effects in AKI by modulating VEGFA-mediated angiogenesis and microcirculatory repair mechanisms.

Luteolin, a natural compound with diverse pharmacological activities, exhibits renoprotective effects against sepsis, renal ischemia, and nephrotoxic agent-induced kidney injury (52). Its protective role is primarily attributed to the inhibition of the NF-κB signaling pathway, thereby attenuating renal fibrosis (53). Quercetin, on the other hand, protects renal tubular epithelial cells from oxidative damage by scavenging oxygen free radicals and suppressing lipid peroxidation (54). β-Carotene, a potent antioxidant, has been shown to significantly reduce arsenic-induced nephrotoxicity, a mechanism linked to its inhibition of the mitochondrial apoptotic pathway and maintenance of redox homeostasis (55).

These findings indicate that BYHWD synergistically targets VEGFA through multiple active components, coordinately regulating oxidative stress, inflammatory responses, and microcirculatory dysfunction. This highlights the therapeutic advantage of TCM formulas in their “multi-component, multi-target, multi-pathway” integrated regulatory approach. The results provide experimental evidence elucidating the molecular mechanisms of BYHWD in AKI treatment and establish a theoretical foundation for future drug development targeting the PI3K-AKT pathway.

### 4.4 Regulatory effects of BYHWD on endothelial cell survival and apoptosis

In this study, the CCK-8 assay demonstrated that BYHWD-medicated serum significantly promoted the proliferation of HUVECs in a dose-dependent manner, with optimal efficacy observed at a 10% concentration (Figure 4A). Trypan blue staining and Annexin V/PI double staining further revealed that BYHWD reduced H/R-induced cell death (Figures 4B, 5A) and inhibited the mitochondrial apoptotic pathway by upregulating the anti-apoptotic protein Bcl-2 and downregulating the pro-apoptotic protein Bax (Figure 7A). These findings align closely with previously reported anti-apoptotic mechanisms of TCM components. For instance, Miao et al. found that quercetin (a primary component of BYHWD) alleviates renal IRI by activating the PI3K/AKT pathway, suppressing Bax activation, and enhancing Bcl-2 expression (56). Similarly, astragaloside IV, derived from *Astragalus membranaceus*, has been shown to inhibit high glucose-induced apoptosis and inflammation in HUVECs by suppressing the JNK signaling pathway (56). Liu et al. reported that BYHWD-medicated serum protects neurons against hypoxia-induced apoptosis, consistent with our results. Additionally, luteolin has been shown to mitigate ROS-mediated cellular damage by inhibiting the Nox4/ROS-NF-κB and MAPK signaling pathways (57), suggesting that the anti-apoptotic effects of BYHWD may arise from the synergistic actions of its multi-component system through multi-target regulatory mechanisms.

### 4.5 Inhibitory effects of BYHWD on oxidative stress and inflammatory response

The results of this study demonstrated that H/R injury significantly elevated intracellular ROS levels in HUVECs (Figure 5B), while BYHWD intervention resulted in a dose-dependent reduction in ROS generation. Oxidative stress is a core mechanism in the progression of AKI, as it promotes the release of pro-inflammatory factors such as IL-1β and IL-18 by activating the NLRP3 inflammasome, thereby exacerbating renal tubular injury (58). Concurrently, this study found that BYHWD significantly downregulated the expression of MCP-1 and α-SMA (Figure 7B), indicating its ability to suppress endothelial-mesenchymal transition (EndMT) and inflammatory responses. Previous studies have shown that microvascular endothelial cells exhibit increased MCP-1 expression and α-SMA-positive cell numbers following ischemic-hypoxic injury. Notably, BYHWD effectively inhibited the upregulation of MCP-1, enhanced CD-31 expression, and alleviated vascular endothelial damage, which aligns with our findings (59, 60).

### 4.6 Activation of VEGF/VEGFRII and PI3K/AKT/FOXO1 pathways by BYHWD

VEGFs are highly specific mitogens for endothelial cells, playing central roles in vasculogenesis, angiogenesis, and vascular homeostasis maintenance. These molecules mediate critical biological functions such as endothelial cell survival, proliferation, migration, and neovascularization through paracrine signaling mechanisms, while regulating vascular permeability (61). The primary receptor for VEGF-A, VEGFR-2 (VEGFRII), initiates downstream cascades by phosphorylating ERK, thereby driving endothelial proliferation and angiogenesis (62). Additionally, VEGFR-2 suppresses apoptosis-related protein expression via PI3K/AKT pathway activation, maintaining endothelial viability and modulating vascular permeability (63). This dual signaling mechanism highlights VEGFR-2’s pivotal role in both physiological vascular homeostasis and pathological angiogenesis.

Previous studies demonstrate that PI3K/AKT activation stimulates KMVEC proliferation and promotes peritubular capillary (PTC) expansion (64). FOXO1, a transcription factor, critically regulates AKI, with Klotho deficiency exacerbating tacrolimus-induced renal damage via PI3K/AKT/FOXO1 signaling (65). FOXO1 phosphorylation is primarily mediated by PI3K-dependent AKT activation (14). Upon injury, PI3K/AKT pathway inhibition reduces FOXO1 phosphorylation and triggers downstream apoptosis-related factors like Bcl-2 and Bax. Evidence suggests that PI3K/AKT/FOXO1 signaling regulates microvascular function, where PI3K/AKT activation promotes angiogenesis via FOXO1 phosphorylation (15, 66).

The VEGF/VEGFRII pathway serves as a master regulator of angiogenesis and endothelial repair, promoting endothelial proliferation, migration, and permeability modulation (67). Our Western blot results revealed that BYHWD significantly downregulated VEGFRII and phosphorylated PI3K/AKT/FOXO1 after H/R injury (Figures 7C,D), suggesting its activating effects on both VEGF/VEGFRII and PI3K/AKT/FOXO1 pathways. Lavoz et al. demonstrated that VEGFRII blockade exhibits renal protective effects in type 2 diabetic DKD models, consistent with our findings (67). Further analysis indicates that astragaloside IV in BYHWD may activate VEGFR2’s tyrosine kinase domain, initiating downstream ERK and PI3K/AKT cascades (68). Wang et al. reported that paeoniflorin binds directly to VEGFRII via the PI3K-AKT pathway, restoring autophagy and inhibiting apoptosis to protect podocytes in DKD, aligning with our results (69).

Based on its clinical foundation of “supplementing qi and activating blood circulation,” BYHWD demonstrates multi-target therapeutic potential in the prevention and treatment of AKI. This study confirms that BYHWD synergistically regulates core targets such as TP53, AKT1, VEGFA, and IL-6, alleviating cellular apoptosis, promoting vascular repair, and suppressing inflammatory responses through the PI3K-AKT/FOXO1 and VEGF/VEGFRII signaling pathways. Future clinical translation can advance along two directions: for AKI induced by chemotherapeutic agents such as cisplatin, it could be developed as a prophylactic renal protective agent; for IRI, its pro-angiogenic function could be utilized to accelerate the recovery of renal function. The high-affinity binding of its active components, such as luteolin, with VEGFA provides a foundation for the further development of precisely targeted drugs. Ultimately, evidence-based medical research is needed to establish clinical pathways for the individualized prevention and treatment of AKI using BYHWD.

Despite the systematic investigation of the potential mechanisms by which BYHWD ameliorates AKI in this study, several limitations remain to be addressed in future research. First, the current findings are primarily based on *in vitro* cellular models, underscoring the need for *in vivo* validation through animal experiments and clinical trials to confirm its efficacy and safety within complex physiological environments. Furthermore, the synergistic or antagonistic interactions among the multiple components of BYHWD and their respective contributions to the overall therapeutic effect require further elucidation. Finally, the exploration of unknown targets and pathways should be enhanced by integrating multi-omics technologies, such as proteomics and metabolomics, to comprehensively uncover additional underlying mechanisms of action.

In summary, H/R injury induces endothelial apoptosis, impairing cell survival, enhancing endothelial-mesenchymal transition (EndMT), and causing insufficient angiogenesis and microcirculatory dysfunction. BYHWD-containing serum mitigates HUVEC damage post-H/R by activating VEGFRII and downstream PI3K/AKT/FOXO1 pathways. Lieberthal et al. found that PI3K/AKT activation protects renal tubular cells from stress-induced apoptosis, ameliorating ischemic AKI. Ruan et al. showed that sika deer antler protein (SDAPR) inhibits FOXO1 via PI3K/AKT signaling to counteract acetaminophen-induced oxidative stress and apoptosis, further corroborating our conclusions.

## 5 Conclusion

This study systematically elucidated the multi-target synergistic mechanism of BYHWD in treating AKI through integrated network pharmacology, molecular docking, and *in vitro* experiments. Network pharmacology analysis revealed that the core components of BYHWD, luteolin and quercetin, bind with high affinity to key target proteins such as VEGFA and AKT1, significantly modulating the phosphorylation cascade of the PI3K-AKT signaling pathway. These components concurrently mediate inflammatory responses and oxidative stress, thereby ameliorating glomerular filtration dysfunction and renal tubular epithelial cell apoptosis. *In vitro* experiments further validated that BYHWD-containing serum alleviates H/R-induced endothelial cell injury via multi-pathway synergy: it activates the VEGF/VEGFRII signaling axis to promote endothelial proliferation and angiogenesis, while suppressing ROS-mediated oxidative stress and MCP-1/α-SMA-associated inflammatory responses. Additionally, it dynamically regulates the PI3K/AKT/FOXO1 pathway to balance cell survival and apoptosis. This research not only deciphers the multi-dimensional therapeutic mode of TCM formulas from the “component-target-pathway” network perspective but also experimentally corroborates the scientific rationale of the TCM theory of “Yi qi Huo xue Tongluo” (invigorating qi, activating blood circulation, and dredging collaterals). It provides dual molecular-level validation (theoretical prediction and experimental evidence) for the clinical application of BYHWD. Future studies should focus on *in vivo* experiments and clinical translational research to advance the modernized application of this formula in AKI therapy.

## Data Availability

The raw data supporting the conclusions of this article will be made available by the authors, without undue reservation.

## References

[1] Kidney Disease: Improving Global Outcomes (KDIGO) CKD Work Group. KDIGO 2024 clinical practice guideline for the evaluation and management of chronic kidney disease. *Kidney Int.* (2024) 105:S117–314. 10.1016/j.kint.2023.10.018 38490803

[2] Hoste EA Bagshaw SM Bellomo R Cely CM Colman R Cruz DN Epidemiology of acute kidney injury in critically ill patients: the multinational AKI-EPI study. *Intensive Care Med.* (2015) 41:1411–23. 10.1007/s00134-015-3934-7 26162677

[3] Ostermann M Lumlertgul N Jeong R See E Joannidis M James M. Acute kidney injury. *Lancet.* (2025) 405:241–56. 10.1016/s0140-6736(24)02385-7 39826969

[4] Susantitaphong P Cruz DN Cerda J Abulfaraj M Alqahtani F Koulouridis I World incidence of AKI: a meta-analysis. *Clin J Am Soc Nephrol.* (2013) 8:1482–93. 10.2215/cjn.00710113 23744003 PMC3805065

[5] Cao F Li Y Peng T Li Y Yang L Hu L PTEN in kidney diseases: a potential therapeutic target in preventing AKI-to-CKD transition. *Front Med (Lausanne).* (2024) 11:1428995. 10.3389/fmed.2024.1428995 39165377 PMC11333338

[6] Li XJ Suo P Wang YN Zou L Nie XL Zhao YY Arachidonic acid metabolism as a therapeutic target in AKI-to-CKD transition. *Front Pharmacol.* (2024) 15:1365802. 10.3389/fphar.2024.1365802 38523633 PMC10957658

[7] Agarwal A Dong Z Harris R Murray P Parikh SM Rosner MH Cellular and molecular mechanisms of AKI. *J Am Soc Nephrol.* (2016) 27:1288–99. 10.1681/asn.2015070740 26860342 PMC4849836

[8] Linkermann A Chen G Dong G Kunzendorf U Krautwald S Dong Z. Regulated cell death in AKI. *J Am Soc Nephrol.* (2014) 25:2689–701. 10.1681/asn.2014030262 24925726 PMC4243360

[9] Ferenbach DA Bonventre JV. Mechanisms of maladaptive repair after AKI leading to accelerated kidney ageing and CKD. *Nat Rev Nephrol.* (2015) 11:264–76. 10.1038/nrneph.2015.3 25643664 PMC4412815

[10] Sun D Feng J Dai C Sun L Jin T Ma J Role of peritubular capillary loss and hypoxia in progressive tubulointerstitial fibrosis in a rat model of aristolochic acid nephropathy. *Am J Nephrol.* (2006) 26:363–71. 10.1159/000094778 16873992

[11] Liu BC Tang TT Lv LL Lan HY. Renal tubule injury: a driving force toward chronic kidney disease. *Kidney Int.* (2018) 93:568–79. 10.1016/j.kint.2017.09.033 29361307

[12] Kida Y Tchao BN Yamaguchi I. Peritubular capillary rarefaction: a new therapeutic target in chronic kidney disease. *Pediatr Nephrol.* (2014) 29:333–42. 10.1007/s00467-013-2430-y 23475077 PMC3726573

[13] Ebert T Tran N Schurgers L Stenvinkel P Shiels PG. Ageing - oxidative stress. PTMs and disease. *Mol Aspects Med.* (2022) 86:101099. 10.1016/j.mam.2022.101099 35689974

[14] Jin J Jin L Lim SW Yang CW. Klotho deficiency aggravates tacrolimus-induced renal injury via the phosphatidylinositol 3-kinase-Akt-forkhead box protein O pathway. *Am J Nephrol.* (2016) 43:357–65. 10.1159/000446447 27174564

[15] Ke J Wei R Yu F Zhang J Hong T. Liraglutide restores angiogenesis in palmitate-impaired human endothelial cells through PI3K/Akt-Foxo1-GTPCH1 pathway. *Peptides.* (2016) 86:95–101. 10.1016/j.peptides.2016.10.009 27777063

[16] Zhang D Jiang H Yang X Zheng S Li Y Liu S Traditional Chinese Medicine and renal regeneration: experimental evidence and future perspectives. *Chin Med.* (2024) 19:77. 10.1186/s13020-024-00935-9 38831435 PMC11149241

[17] Fu X Sun Z Long Q Tan W Ding H Liu X Glycosides from Buyang Huanwu Decoction inhibit atherosclerotic inflammation via JAK/STAT signaling pathway. *Phytomedicine.* (2022) 105:154385. 10.1016/j.phymed.2022.154385 35987015

[18] Liu B Song Z Yu J Li P Tang Y Ge J. The atherosclerosis-ameliorating effects and molecular mechanisms of BuYangHuanWu decoction. *Biomed Pharmacother.* (2020) 123:109664. 10.1016/j.biopha.2019.109664 31887542

[19] Han X Zhang G Chen G Wu Y Xu T Xu H Buyang Huanwu Decoction promotes angiogenesis in myocardial infarction through suppression of PTEN and activation of the PI3K/Akt signalling pathway. *J Ethnopharmacol.* (2022) 287:114929. 10.1016/j.jep.2021.114929 34952189

[20] Chen Y Hu Z Li Y Lan X Bai Y Long H Pharmacologically active constituents of Buyang Huanwu Decoction against cerebral ischemia-reperfusion injury and verification of its effects on oxidative stress, energy metabolism, and inflammation. *J Ethnopharmacol* (2025) 353:120405. 10.1016/j.jep.2025.120405 40835193

[21] Luo Y Hu W Li Z Zhang X Chen S Yang Q Integrating network pharmacology and in vivo validation to explore the mechanisms of Buyang Huanwu Decoction in myocardial ischemia-reperfusion injury. *J Inflamm Res.* (2025) 18:7493–514. 10.2147/jir.S512050 40524966 PMC12169005

[22] Zhu M Wei J Li Y Wang Y Ren J Li B Efficacy and mechanism of Buyang Huanwu Decoction in patients with ischemic heart failure: a randomized, double-blind, placebo-controlled trial combined with proteomic analysis. *Front Pharmacol.* (2022) 13:831208. 10.3389/fphar.2022.831208 35370712 PMC8971676

[23] Liu Y Huang Y Sun D Ye N Chen T Yang M Research progress of astragaloside IV in treating acute kidney injury. *Int Urol Nephrol.* (2024) 56:2645–50. 10.1007/s11255-024-04016-6 38494585

[24] Tang JL Xin M Zhang LC. Protective effect of *Astragalus membranaceus* and astragaloside IV in sepsis-induced acute kidney injury. *Aging (Albany NY).* (2022) 14:5855–77. 10.18632/aging.204189 35859295 PMC9365550

[25] Shi J Li S Yi L Gao M Cai J Yang C Levistolide a attenuates acute kidney injury in mice by inhibiting the TLR-4/NF-κB pathway. *Drug Des Devel Ther.* (2024) 18:5583–97. 10.2147/dddt.S476548 39654604 PMC11625643

[26] Zhang MY Ma LJ Jiang L Gao L Wang X Huang YB Paeoniflorin protects against cisplatin-induced acute kidney injury through targeting Hsp90AA1-Akt protein-protein interaction. *J Ethnopharmacol.* (2023) 310:116422. 10.1016/j.jep.2023.116422 36972781

[27] Xing D Ma Y Lu M Liu W Zhou H. Paeoniflorin alleviates hypoxia/reoxygenation injury in HK-2 cells by inhibiting apoptosis and repressing oxidative damage via Keap1/Nrf2/HO-1 pathway. *BMC Nephrol.* (2023) 24:314. 10.1186/s12882-023-03366-0 37884904 PMC10601317

[28] Bunel V Antoine MH Nortier J Duez P Stévigny C. Potential nephroprotective effects of the Chinese herb Angelica sinensis against cisplatin tubulotoxicity. *Pharm Biol.* (2015) 53:985–94. 10.3109/13880209.2014.951726 25495691

[29] Wang Y Han K Li Z Tang X Wang C Zhao Y Protective effect of hydroxysafflor yellow A on renal ischemia–reperfusion injury by targeting the Akt-Nrf2 axis in mice. *Exp Ther Med.* (2022) 24:741. 10.3892/etm.2022.11677 36478883 PMC9716340

[30] Wang RY Jia D Zhang WQ Wu H Yang T Xu T Hydroxysafflower yellow A mitigates renal ischemia-reperfusion injury by inhibiting the CCR4-mediated apoptosis pathway. *Phytomedicine.* (2025) 143:156857. 10.1016/j.phymed.2025.156857 40446579

[31] Cao JY Wang B Tang TT Wen Y Li ZL Feng ST Exosomal miR-125b-5p deriving from mesenchymal stem cells promotes tubular repair by suppression of p53 in ischemic acute kidney injury. *Theranostics.* (2021) 11:5248–66. 10.7150/thno.54550 33859745 PMC8039965

[32] Ding Y Zhou DY Yu H Zhu T Guo F He Y Upregulation of lncRNA NONRATG019935.2 suppresses the p53-mediated apoptosis of renal tubular epithelial cells in septic acute kidney injury. *Cell Death Dis.* (2021) 12:771. 10.1038/s41419-021-03953-9 34719669 PMC8558325

[33] Lv W Liao J Li C Liu D Luo X Diao R Aquaporin 1 is renoprotective in septic acute kidney injury by attenuating inflammation, apoptosis and fibrosis through inhibition of P53 expression. *Front Immunol.* (2024) 15:1443108. 10.3389/fimmu.2024.1443108 39238634 PMC11374652

[34] Sun M Li J Mao L Wu J Deng Z He M p53 deacetylation alleviates sepsis-induced acute kidney injury by promoting autophagy. *Front Immunol.* (2021) 12:685523. 10.3389/fimmu.2021.685523 34335587 PMC8318785

[35] Kim IY Lee MY Park MW Seong EY Lee DW Lee SB Deletion of Akt1 promotes kidney fibrosis in a murine model of unilateral ureteral obstruction. *Biomed Res Int.* (2020) 2020:6143542. 10.1155/2020/6143542 33299873 PMC7707954

[36] Hu J Bu W Ding Y Li X Zhang B Shen B Jian Pi Hua Tan Fang reverses trastuzumab resistance of HER2-positive gastric cancer through PI3K/AKT/mTOR pathway: integrating network pharmacology, molecular docking and experimental validation. *Immun Inflamm Dis.* (2025) 13:e70154. 10.1002/iid3.70154 39917999 PMC11803458

[37] Xu C Deng Y Man J Wang H Che T Ding L Unveiling the renoprotective mechanisms of schisandrin B in ischemia-reperfusion injury through transcriptomic and pharmacological analysis. *Drug Des Devel Ther.* (2024) 18:4241–56. 10.2147/dddt.S489458 39323973 PMC11423835

[38] Rotllan N Wanschel AC Fernández-Hernando A Salerno AG Offermanns S Sessa WC Genetic evidence supports a major role for Akt1 in VSMCs during atherogenesis. *Circ Res.* (2015) 116:1744–52. 10.1161/circresaha.116.305895 25868464 PMC4561531

[39] Huang MJ Ji YW Chen JW Li D Zhou T Qi P Targeted VEGFA therapy in regulating early acute kidney injury and late fibrosis. *Acta Pharmacol Sin.* (2023) 44:1815–25. 10.1038/s41401-023-01070-1 37055531 PMC10462693

[40] Zhong X Tang TT Shen AR Cao JY Jing J Wang C Tubular epithelial cells-derived small extracellular vesicle-VEGF-A promotes peritubular capillary repair in ischemic kidney injury. *NPJ Regen Med.* (2022) 7:73. 10.1038/s41536-022-00268-x 36528739 PMC9759551

[41] Sánchez-Navarro A Pérez-Villalva R Murillo-de-Ozores AR Martínez-Rojas M Rodríguez-Aguilera JR González N Vegfa promoter gene hypermethylation at HIF1α binding site is an early contributor to CKD progression after renal ischemia. *Sci Rep.* (2021) 11:8769. 10.1038/s41598-021-88000-5 33888767 PMC8062449

[42] Andres-Hernando A Okamura K Bhargava R Kiekhaefer CM Soranno D Kirkbride-Romeo LA Circulating IL-6 upregulates IL-10 production in splenic CD4(+) T cells and limits acute kidney injury-induced lung inflammation. *Kidney Int.* (2017) 91:1057–69. 10.1016/j.kint.2016.12.014 28214022

[43] Chen J Wang W Tang Y Huang XR Yu X Lan HY. Inflammatory stress in SARS-COV-2 associated acute kidney injury. *Int J Biol Sci.* (2021) 17:1497–506. 10.7150/ijbs.58791 33907513 PMC8071761

[44] Chen Y Zhou X Wu Y. The miR-26a-5p/IL-6 axis alleviates sepsis-induced acute kidney injury by inhibiting renal inflammation. *Ren Fail.* (2022) 44:551–61. 10.1080/0886022x.2022.2056486 35491874 PMC9067948

[45] Guo X Zhu Y Sun Y Li X. IL-6 accelerates renal fibrosis after acute kidney injury via DNMT1-dependent FOXO3a methylation and activation of Wnt/β-catenin pathway. *Int Immunopharmacol.* (2022) 109:108746. 10.1016/j.intimp.2022.108746 35569307

[46] Wang J Hu K Cai X Yang B He Q Wang J Targeting PI3K/AKT signaling for treatment of idiopathic pulmonary fibrosis. *Acta Pharm Sin B.* (2022) 12:18–32. 10.1016/j.apsb.2021.07.023 35127370 PMC8799876

[47] Wang M Zhang J Gong N. Role of the PI3K/Akt signaling pathway in liver ischemia reperfusion injury: a narrative review. *Ann Palliat Med.* (2022) 11:806–17. 10.21037/apm-21-3286 35016518

[48] Chen J Zhang X Liu X Zhang C Shang W Xue J Ginsenoside Rg1 promotes cerebral angiogenesis via the PI3K/Akt/mTOR signaling pathway in ischemic mice. *Eur J Pharmacol.* (2019) 856:172418. 10.1016/j.ejphar.2019.172418 31132356

[49] Chen L Qin L Liu X Meng X. CTRP3 alleviates Ox-LDL-induced inflammatory response and endothelial dysfunction in mouse aortic endothelial cells by activating the PI3K/Akt/eNOS pathway. *Inflammation.* (2019) 42:1350–9. 10.1007/s10753-019-00996-1 30887395

[50] Sanajou D Ghorbani Haghjo A Argani H Aslani S. AGE-RAGE axis blockade in diabetic nephropathy: current status and future directions. *Eur J Pharmacol.* (2018) 833:158–64. 10.1016/j.ejphar.2018.06.001 29883668

[51] Yiu WH Wong DW Wu HJ Li RX Yam I Chan LY Kallistatin protects against diabetic nephropathy in db/db mice by suppressing AGE-RAGE-induced oxidative stress. *Kidney Int.* (2016) 89:386–98. 10.1038/ki.2015.331 26536000

[52] Diniz LRL Elshabrawy HA Souza MTS Duarte ABS Madhav N de Sousa DP. Renoprotective effects of luteolin: therapeutic potential for COVID-19-associated acute kidney injuries. *Biomolecules.* (2022) 12:1544. 10.3390/biom12111544 36358895 PMC9687696

[53] Oyagbemi AA Akinrinde AS Adebiyi OE Jarikre TA Omobowale TO Ola-Davies OE Luteolin supplementation ameliorates cobalt-induced oxidative stress and inflammation by suppressing NF-κB/Kim-1 signaling in the heart and kidney of rats. *Environ Toxicol Pharmacol.* (2020) 80:103488. 10.1016/j.etap.2020.103488 32898663

[54] Renugadevi J Prabu SM. Quercetin protects against oxidative stress-related renal dysfunction by cadmium in rats. *Exp Toxicol Pathol.* (2010) 62:471–81. 10.1016/j.etp.2009.06.006 19615871

[55] Das R Das A Roy A Kumari U Bhattacharya S Haldar PK. β-Carotene ameliorates arsenic-induced toxicity in albino mice. *Biol Trace Elem Res.* (2015) 164:226–33. 10.1007/s12011-014-0212-4 25542264

[56] Miao Z Miao Z Wang S Shi X Xu S. Quercetin antagonizes imidacloprid-induced mitochondrial apoptosis through PTEN/PI3K/AKT in grass carp hepatocytes. *Environ Pollut.* (2021) 290:118036. 10.1016/j.envpol.2021.118036 34488159

[57] Liu Z Yin M Li J Wang J Jin X Zhou X Buyang Huanwu Decoction restores the balance of mitochondrial dynamics after cerebral ischemia-reperfusion through calcium overload reduction by the PKCε-Nampt-Sirt5 axis. *J Ethnopharmacol.* (2025) 338:119003. 10.1016/j.jep.2024.119003 39528118

[58] Xia F Wang C Jin Y Liu Q Meng Q Liu K Luteolin protects HUVECs from TNF-α-induced oxidative stress and inflammation via its effects on the Nox4/ROS-NF-κB and MAPK pathways. *J Atheroscler Thromb.* (2014) 21:768–83. 10.5551/jat.23697 24621786

[59] Mulay SR Honarpisheh MM Foresto-Neto O Shi C Desai J Zhao ZB Mitochondria permeability transition versus necroptosis in oxalate-induced AKI. *J Am Soc Nephrol.* (2019) 30:1857–69. 10.1681/asn.2018121218 31296606 PMC6779355

[60] Li Y Miao L Guo R He L Sun M Pan Y To explore the regulatory effect of Buyang Huanwu Decoction on cerebral infarction based on quantitative proteomics. *J Proteomics.* (2023) 277:104850. 10.1016/j.jprot.2023.104850 36813112

[61] Chakraborty A Chatterjee M Twaalfhoven H Del Campo Milan M Teunissen CE Scheltens P Vascular Endothelial Growth Factor remains unchanged in cerebrospinal fluid of patients with Alzheimer’s disease and vascular dementia. *Alzheimers Res Ther.* (2018) 10:58. 10.1186/s13195-018-0385-8 29933741 PMC6015445

[62] Cho HD Moon KD Park KH Lee YS Seo KI. Effects of auriculasin on vascular endothelial growth factor (VEGF)-induced angiogenesis via regulation of VEGF receptor 2 signaling pathways in vitro and in vivo. *Food Chem Toxicol.* (2018) 121:612–21. 10.1016/j.fct.2018.09.025 30236598

[63] Wang X Jiang L Liu XQ Huang YB Wang AL Zeng HX Paeoniflorin binds to VEGFR2 to restore autophagy and inhibit apoptosis for podocyte protection in diabetic kidney disease through PI3K-AKT signaling pathway. *Phytomedicine.* (2022) 106:154400. 10.1016/j.phymed.2022.154400 36049428

[64] Dang LTH Aburatani T Marsh GA Johnson BG Alimperti S Yoon CJ Hyperactive FOXO1 results in lack of tip stalk identity and deficient microvascular regeneration during kidney injury. *Biomaterials.* (2017) 141:314–29. 10.1016/j.biomaterials.2017.07.010 28711779 PMC5567800

[65] He Y Yang X Zhang C Deng M Tu B Liu Q Ablation of macrophage transcriptional factor FoxO1 protects against ischemia-reperfusion injury-induced acute kidney injury. *Acta Pharm Sin B.* (2025) 15:3107–24. 10.1016/j.apsb.2025.04.009 40654361 PMC12254786

[66] Ruan H Luo J Wang L Wang J Wang Z Zhang J. Sika deer antler protein against acetaminophen-induced nephrotoxicity by activating Nrf2 and inhibition FoxO1 via PI3K/Akt signaling. *Int J Biol Macromol.* (2019) 141:961–87. 10.1016/j.ijbiomac.2019.08.164 31479670

[67] Lavoz C Rodrigues-Diez RR Plaza A Carpio D Egido J Ruiz-Ortega M VEGFR2 blockade improves renal damage in an experimental model of type 2 diabetic nephropathy. *J Clin Med* (2020) 9:302. 10.3390/jcm9020302 31973092 PMC7074274

[68] Li YL Lu ZH Zhang YY Wu SS Xie TH Ding H [Mechanism of astragaloside IV combined with Panax notoginseng saponins in regulating angiogenesis to treat cerebral ischemia based on network pharmacology and experimental verification]. *Zhongguo Zhong Yao Za Zhi.* (2024) 49:1017–27. 10.19540/j.cnki.cjcmm.20230901.401 38621909

[69] Lieberthal W Tang M Abate M Lusco M Levine JS. AMPK-mediated activation of Akt protects renal tubular cells from stress-induced apoptosis in vitro and ameliorates ischemic AKI in vivo. *Am J Physiol Renal Physiol.* (2019) 317:F1–11. 10.1152/ajprenal.00553.2018 30995114

